# Comparison of conventional fenestration discectomy with Transforaminal endoscopic lumbar discectomy for treating lumbar disc herniation:minimum 2-year long-term follow-up in 1100 patients

**DOI:** 10.1186/s12891-020-03652-0

**Published:** 2020-09-23

**Authors:** Quanyi Li, Yongchun Zhou

**Affiliations:** Department of Orthopedic, Shaanxi Provincial People’s Hospital, 256# You-yi West Road, Xi’an, 710068 Shaanxi People’s Republic of China

**Keywords:** Posterior approach, Transforaminal endoscopic discectomy, Lumbar disc herniation, Efficacy

## Abstract

**Purpose:**

To compare the efficacy of conventional interlaminar fenestration discectomy (IFD) with transforaminal endoscopic lumbar discectomy (TELD) for treating lumbar disc herniation (LDH).

**Methods:**

The clinical data of 1100 patients who had been diagnosed with LDH between January 2012 and December 2017 were retrospectively analysed. IFD was performed on 605 patients in Group A, whereas TELD was performed on 505 patients in Group B. The Oswestry Disability Index, Visual Analogue Scale for pain and modified MacNab criteria were used to evaluate the outcomes. The surgery duration, intraoperative blood loss, postoperative off-bed activity and postoperative length of hospital stay were recorded.

**Results:**

The follow-up period ranged from 24 to 60 months, with an average of 43 months. The excellent and good outcome rates were 93.5% in Group A and 92.6% in Group B. There was no significant difference in efficacy between the groups (*P* > 0.05). However, Group B had significantly less intraoperative blood loss and shorter bed rest duration and postoperative length of hospital stay than Group A (*P* < 0.05). There were two cases of postoperative recurrence in Group A and three in Group B.

**Conclusions:**

Although conventional IFD and TELD had similar levels of efficacy in treating LDH, TELD had several advantages. There was less intraoperative bleeding, shorter length of hospital stay and shorter bed rest duration. It can be considered a safe and effective surgical option for treating LDH.

## Introduction

Lumbar disc herniation (LDH) is a condition in which the spine deteriorates and herniation of the intervertebral disc occurs. It is commonly observed in orthopaedic clinics and can cause severe symptoms, including lower back pain and sciatica, which greatly impact patients’ daily lives. Most patients are admitted to hospital [1].

Conservative treatment is often preferred for LDH, but patients who fail to respond to this are treated with surgery [2]. Surgical treatment aims to remove the herniated nucleus pulposus to the largest extent possible to relieve nerve compression while minimising spinal instability [3]. Interlaminar fenestration discectomy (IFD) is the most commonly performed surgical procedure for treating LDH and is considered the gold standard [4]. Although the herniated nucleus pulposus can be completely removed to relieve nerve compression, it will affect spinal stability, as it requires partial removal of the posterior portion of the spine [5, 6]. With the development of minimally invasive techniques in recent years, endoscopic lumbar discectomy has attracted the interest of scholars. With transforaminal endoscopic lumbar discectomy (TELD), the degenerated nucleus pulposus can be completely removed. This directly decompresses the nerve root while preserving the anatomy and biomechanical stability of the lumbar spine [7, 8].

Some studies on the therapeutic effects of IFD and TELD on LDH have been conducted [9–11]. A study compared IFD and TELD in the treatment of LDH and reported significantly shorter operation time and hospitalisation time and a lower bleeding volume in the TELD group than in the IFD group [9]. Other studies have shown that TELD is associated with a better postoperative Oswestry Disability Index (ODI) score, lower blood loss and shorter operation time and hospitalisation time. The complications did not differ significantly between TELD and IFD in the treatment of LDH [10, 11]. However, there are still few studies with a large sample size and long-term follow-up. This study was designed to compare the clinical efficacy of conventional IFD versus TELD in treating LDH with a maximum 5 year follow-up in a large population.

## Materials and methods

### Patient population

A total of 1100 patients who had been diagnosed with single-segment LDH by X-ray, computed tomography (CT) and magnetic resonance imaging (MRI) and underwent lumbar discectomy with IFD or TELD between January 2012 and December 2017 in our hospital were included in this study. All patients underwent formal conservative treatment, including bed rest, lumbar traction, physical therapy and oral nonsteroidal anti-inflammatory drugs for 3 months. Patients with an inadequate response to treatment were then treated with surgery. Other inclusion criteria were that the herniation site was L3/4, L4/5 or L5/S1 herniation and that the herniation type was posterolateral, central, paracentral or extreme lateral herniation. The exclusion criteria were as follows: (1) obvious lumbar instability evident on X-ray, (2) central stenosis confirmed by CT or MRI, (3) severe ossification of the posterior longitudinal ligament, (4) large posterior central herniation, (5) unconsciousness or inability to adhere to the treatment, (6) refusal to sign the informed consent form, (7) a lumbar deformity or tumour, (8) infection at the surgical site, and (9) severe liver and kidney dysfunction or cardiovascular or cerebrovascular disease. Patients were divided into two groups: Group A underwent IFD and Group B underwent TELD.

### Operative technique

Each patient in Group A was placed in the prone position under general anaesthesia. A 4–6 cm posterior midline incision was made with the deteriorating segment positioned in the centre. The lumbar fascia was exposed, and the attachment of the spinalis muscle was cut near the spinous process so that the supraspinous and interspinous ligaments were preserved. The soft tissue behind the laminae was stripped to reveal the intervertebral space, upper and lower lamina and small joints. A laminar rongeur was used to remove the ligamentum flavum between the lamina and small portions of the upper and lower lamina adjacent to the deteriorating segment; thus, interlaminar fenestration was performed. A neuroexfoliator was used to separate and gently retract the nerve root, revealing the intervertebral disc. The fibrous ring was cut, and the nucleus pulposus was removed with dedicated forceps. The incision was closed [4, 12].

Each patient in Group B was placed in the lateral recumbent position. C-arm X-ray was used to locate the surface projection of the intervertebral space of interest. An entry point was made 12–14 cm from the posterior midline of the spine at the level of the disc. Local anaesthesia was administered (1% lidocaine). A puncture needle was slowly advanced to the fibrous ring in the intervertebral space and positioned at the outer edge of the superior articular process. It was located lateral to the intervertebral space near the upper edge of the lower vertebra. One millilitre of methylene blue was injected into the intervertebral disc for contrast radiography. A guidewire was inserted, and X-ray was used to confirm that the tip of the guidewire had crossed the articular process and then the puncture needle was withdrawn. An 8 mm incision was made at the entry point. The cannulas were passed from thin to thick along the guidewire, and the superior articular process was partially removed with a ring drill. The working cannula was then inserted into the epidural space. A transforaminal endoscope (TESSYS® [transforaminal endoscopic spine system], joimax® GmBH, Germany) was inserted, and the degenerative, blue-stained intervertebral disc was removed. Part of the nucleus pulposus was ablated by a radiofrequency electrode (Ellman, USA). The spinal canal was assessed carefully, and the nerve root was detached. After the wound was rinsed, the fenestrated fibrous ring was repaired by electrocoagulation. The working cannula was then removed, and the incision was closed [8].

### Postoperative care

Patients were asked to perform straight leg raises in bed on the same day after surgery, and off-bed training with lower back braces was initiated 2 days later for Group A patients, or 1 day later for Group B patients.

### Evaluation measures

Patients were asked to use the Visual Analogue Scale (VAS) [13] to rate the severity of the pain in their lower back and legs before surgery, 1 month after and at the final follow-up appointment. A score of 0 points corresponded to no pain; 1 to 3 points, to slight pain; 4 to 6 points, to obvious pain that affected sleep but was still tolerable and 7 to 10 points, to intense, unbearable pain. Functional changes were evaluated using the ODI [14], which has 10 questions on the severity of pain, ability to perform self-care, lifting objects, walking, sitting, standing, sleeping, social life and travel. There are six response options for each question, and the highest score for each question is 5 points. The lower the score was, the better the postoperative recovery. The MacNab criteria [15] were used to evaluate surgical efficacy. Patient outcomes were graded as excellent, good, fair or poor, representing no symptoms, mild symptoms and slight limitations in mobility, improved symptoms but large limitations in mobility and unimproved or even worsened symptoms, respectively. The excellent and good rate was calculated as follows: (excellent + good)/total cases × 100%.

### Statistical analysis

SPSS 19.0 (SPSS, IL, USA) software was used for the statistical analyses. Measurement data are expressed as the mean ± standard deviation (x ± s). Comparisons between groups were performed by one-way analysis of variance and t-tests. Comparisons of the count data, as well as the ‘excellent’ and ‘good’ rates between the two groups, were performed using the *x*^2^ test. *P* < 0.05 indicated a significant difference.

## Results

A total of 1100 patients were included in this study. In Group A, 605 patients who underwent conventional IFD were included, and in Group B, 505 patients who underwent TELD were included. In Group A, there were 300 males and 305 females, with a mean age of 42.9 ± 12.4 (ranging from 23 to 64) years old. In Group B, there were 285 males and 220 females, with a mean age of 40.5 ± 13.7 (ranging from 20 to 67) years old. The difference in surgery duration between the two groups was not statistically significant (*P* > 0.05). However, Group B had significantly less intraoperative blood loss and shorter bed rest duration and postoperative length of hospital stay than Group A (*P* < 0.05). The surgical characteristics of the patients are summarised in Table 1.

**Table 1:** Surgical characteristics of patients in the two groups

Characteristic	Group A (***n*** = 605)	Group B (***n*** = 505)
Sex, males (%)	300 (49.6%)	285 (56.4%)
Age at initial operation (years)	42.9 ± 12.4 (23–64)	40.5 ± 13.7 (20–67)
Posterolateral herniation	224 (37.0%)	196 (38.8%)
Central herniation	78 (12.9%)	50 (9.9%)
Paracentral herniation	218 (36.0%)	206 (40.8%)
Extreme lateral herniation	85 (14.1%)	53 (10.5%)
L3/4 herniation	136 (22.4%)	107 (21.2%)
L4/5 herniation	252 (41.7%)	220 (43.6%)
L5/S1 herniation	217 (35.9%)	178 (35.2%)
Surgery duration (min)^*^	65.5 ± 6.0 (42–113)	63.6 ± 6.3 (40–108)
Intraoperative blood loss (ml)^*^	80 ± 10 (50–120)	15.3 ± 11 (3–40)
Length of hospital stay (d)^*^	7.3 ± 0.9 (6–11)	4.3 ± 0.3 (2–8)
Bed rest duration (d)^*^	3.23 ± 0.5 (1–6)	1.6 ± 0.4 (1–3)

Data are denoted as *n* (%) or mean ± standard deviation (range)

^*^P < 0.05 for Group A vs. Group B

All patients were followed up for 26 to 60 months after surgery, with a mean duration of 43.67 ± 7.0 months. Three cases of surgical complications occurred in Group A: one wound infection, which healed after antibiotic treatment, and two cases of cerebrospinal fluid leakage. During follow-up, one case recurred 11 months after surgery and another 18 months after. Both cases were resolved by decompressive laminectomy via the posterior approach and internal fixation with an intervertebral fusion cage. In Group B, there were four cases of dura mater injury and, thus, cerebrospinal fluid leakage. During the follow-up period, there were three recurrences, which occurred at 11, 16 and 26 months after surgery. Two were resolved by repeat TELD, and one was cured by decompressive laminectomy via the posterior approach and internal fixation with an intervertebral fusion cage. Neither group had complications such as nerve root or cauda equina injury.

There was no statistically significant difference in the VAS scores for leg pain between the two groups (*P* > 0.05), and the scores progressively decreased. There was no significant difference in the VAS scores for back pain scores between the groups 1 month after surgery or at the last follow-up. One day after surgery, however, the VAS scores were significantly higher in Group A than in Group B (*P* < 0.05) (Table 2). One day after surgery, 1 month after surgery and at the last follow-up, there was no significant difference in the ODI values between the two groups (*P >* 0.05). The values in both groups were significantly lower than those before surgery (*P* < 0.05) (Table 2).

**Table 2:** Measures of baseline severity and surgical outcomes in the two groups

Measure	Group A	Group B
VAS-LP		
Preoperative	7.1 ± 1.3	7.2 ± 1.2
1-day postoperative	2.0 ± 0.9^*^	1.9 ± 0.8^*^
1-month postoperative	1.8 ± 0.4^Δ^	1.7 ± 0.5^Δ^
Last follow-up	0.6 ± 0.07^+^	0.6 ± 0.08^+^
VAS-BP		
Preoperative	7.9 ± 1.0	8.1 ± 1.1
1-day postoperative	4.4 ± 0.8^*^	2.8 ± 0.9^*#^
1-month postoperative	2.0 ± 0.5^Δ^	1.9 ± 0.6^Δ^
Last follow-up	0.7 ± 0.04^+^	0.7 ± 0.05^+^
ODI, %		
Preoperative	68.3 ± 14.1	69.2 ± 12.2
1-day postoperative	24.2 ± 3.1^*^	23.9 ± 3.2^*^
1-month postoperative	15.6 ± 0.1^Δ^	16.1 ± 0.2^Δ^
Last follow-up	7.9 ± 0.04^+^	8.3 ± 0.03^+^

*ODI* Oswestry Disability Index, *VAS-LP* Visual Analogue Scale for Leg Pain, *VAS-BP* Visual Analogue Scale for Back Pain

*P < 0.05 vs. preoperative

^Δ^*P* < 0.05 vs. 1-day postoperative

^+^*P* < 0.05 vs. 1-month postoperative

^#^*P* < 0.05, Group A vs. Group B

According to the modified MacNab criteria, in Group A, the outcomes were excellent in 483 cases (79.8%), good in 88 cases (14.6%) and fair in 36 cases (5.6%), and the excellent and good rate was 93.5%. In Group B, the outcomes were excellent in 392 cases (77.6%), good in 86 cases (17.0%) and fair in 27 cases (5.4%), and the excellent and good rate was 92.6%. The outcomes of both groups were tested by *x*^2^, and there was no significant difference (*P* > 0.05).

## Discussion

Patients who fail to respond to conservative treatment are treated with surgery. The aim is to remove the herniated nucleus pulposus and relieve nerve compression while minimising spinal instability [1–4]. IFD is the most commonly performed operation and is considered the gold standard procedure for treating LDH. The operation is technically easy and offers a clear field of vision. The surgeons can reveal and cut the ligamentum flavum and the pathological bone hyperplasia under direct observation to expand and decompress the nerve root canals. The normal anatomy of the spine is preserved to the greatest extent possible to ensure that patients can undergo early postoperative rehabilitation [5, 6].

Nevertheless, in clinical practice, since the nerve root and dural sac need to be retracted to expose the disc, the dura can easily be injured, and there is a high risk of adherent or damaged nerve roots. Moreover, the soft tissue needs to be stripped during this surgery, which can lead to denervation of the muscles. This is not conducive to postoperative recovery [16, 17]. In this study, there were two cases of dura mater injury complicated with cerebrospinal fluid leakage. One day after surgery, the severity of lower back pain was significantly higher in Group A than in Group B, which might be related to IFD, causing more trauma.

In recent years, many advances in spinal endoscopy have been made, and lumbar discectomy under endoscopy is frequently performed for the treatment of LDH. Transforaminal endoscopic discectomy can be performed to remove the herniated nucleus pulposus directly via the subforaminal safe-triangle approach to reduce the central pressure on the intervertebral disc. It also minimises damages to the tissues and maintains spinal stability [6, 7]. Combined with radiofrequency bipolar haemostasis and reconstruction of the fenestrated fibrous ring, this surgical approach greatly reduces the amount of postoperative scarring around the nerve root. It also reduces the severity of denervation in the ablation of the intervertebral disc and alleviates the postoperative symptoms of lower back pain [18, 19]. Radiating pain in the lower limbs is caused by mechanical compression and chemical stimulation at the nerve root. After the nucleus pulposus has been removed, the centre of the intervertebral disc is decompressed, allowing the fibrous ring, especially its herniated portion, to retract, which is the first stage of decompression of the nerve root. When the tongue-shaped end of the working cannula is retracted near the lateral recess, the course of the nerve root and the side of the dural sac can be identified by rotating and turning the cannula. The adhesions and un-retracted bulges and herniations can be removed directly under the endoscope, achieving the second stage of local decompression of the nerve root. Ring drills used in TELD can be used to enlarge the transforaminal working cannula to a moderate extent. This allows the endoscope to reach any position inside the intervertebral disc and the spinal canal on the affected side to remove the herniated tissue. The shortcomings of incomplete decompression after early transforaminal endoscopic discectomy are thus entirely resolved. After intraoperative injection of methylene blue into the intervertebral disc, the degenerative and damaged tissues are first stained with dark blue. The nerve root, the fibrous ring and the dural sac are not stained, for the most part, thereby improving the surgeon’s ability to identify the tissue to be removed.

Compared with IFD, TELD involves a smaller incision and a clearer field of vision, whereas the ligamentum flavum and the paravertebral muscles are not affected. This reduces the extent of postoperative adhesions and denervation of the nerve root inside the spinal canal. Moreover, the surgery is done under local anaesthesia, which enables communication with the patient during the procedure to prevent nerve damage. Cannulas of various sizes are used to establish the working cannula, thereby minimising damage to the spine and ensuring lumbar spinal stability [20, 21].

This study showed that TELD leads to less intraoperative blood loss, faster off-bed rehabilitation and a shorter length of hospital stay, which is consistent with other results. It also showed no statistically significant difference in surgery duration between the two groups. This may be because the surgeons’ expertise in minimally invasive techniques is constantly improving.

Different spine surgical options have their advantages. The surgical method should be selected according to the type of herniated disc to achieve the optimal surgical outcome, minimise postoperative complications and improve the patient’s experience.

This study had some limitations: it was a single-centre retrospective study design, and the patient population was not very homogenous. In the future, a prospective study will be conducted to analyse the efficacy of IFD and TELD in treating LDH.

## Conclusion

Given the appropriate surgical indications, both IFD and TELD can be used to achieve desirable outcomes in the treatment of LDH. However, compared with IFD, TELD exhibited several advantages, such as less bleeding and a shorter length of hospital stay and bed rest duration. It can be considered an ideal surgical option for treating LDH.

## Data Availability

The datasets supporting the conclusions of this article are included within the article. The raw data can be requested from the corresponding author upon reasonable request.

## Abbreviations

IFD: Interlaminar fenestration discectomy
TELD: transforaminal endoscopic lumbar discectomy
CT: Computed tomography
MRI: Magnetic resonance imaging
VAS: Visual analog scale
ODI: Oswestry Disability Index
Post-op: post-operation
VAS-LP: Visual Analogue Scale for Leg Pain
VAS-BP: Visual Analogue Scale for Back Pain
